# A review of current evidence and perspectives on the mechanisms and clinical significance of hypoxia-induced remodeling of the gastric microbiota-metabolism axis

**DOI:** 10.3389/fmicb.2026.1846664

**Published:** 2026-06-23

**Authors:** YiWen Zhang, Zhe Yuan

**Affiliations:** Department of Gastroenterology, Affiliated Hospital of Qinghai University Affiliated Hospital, Xining, China

**Keywords:** gastric diseases, gastric microbiota, hypoxia, metabolism axis, metabolomics, microecological remodeling

## Abstract

Hypoxia, as a critical environmental factor, significantly influences the gastric microbiota. The microbiota-metabolism axis profoundly influences host health and disease through its effects on microbial composition and metabolic processes. This review examines how gastric hypoxia affects microbial populations in distinct ways and how these metabolic changes may contribute to gastric disorders. Integrating recent insights from molecular biology and metabolomics, we elucidate the mechanisms underlying microbial dysbiosis in hypoxic environments and their impact on downstream signaling pathways. These findings implicate this axis in the pathogenesis of gastritis, gastric ulcers, and gastric cancer. Finally, we discuss key considerations for future clinical implementation, acknowledging that the current evidence base remains largely observational and indirect. Moreover, we offer a novel perspective on the interplay between hypoxia, bacteria, and metabolism within the gastric niche.

## 1 Introduction

Hypoxia is a critical environmental factor that affects the physiology and pathophysiology of the stomach lining. The gastric mucosa exhibits a distinctive oxygen landscape shaped by its unique hemodynamic and metabolic features. Inflammation, infection, or neoplasia can exacerbate mucosal hypoxia. These outcomes arise from the modulation of cellular function, immune responses, and barrier integrity. HIF-1α and HIF-2α act as pivotal molecular switches that initiate adaptive responses, such as altering metabolism toward glycolysis, modulating reactive oxygen species (ROS), and initiating inflammatory pathways. For instance, HIF-1α activates Drp1, leading to mitochondrial fission. This increases ROS levels and activates the NOD-like receptor protein 3 (NLRP3) inflammasome, worsening mucosal damage (Xiao et al., 2024). Hypoxia increases programmed death-ligand 1 (PD-L1) levels via the FOXO3a pathway, compromising immune surveillance and reducing the efficacy of immunotherapy (Xiang et al., 2025). These findings underscore the centrality of hypoxia in gastric mucosal biology, particularly in the context of antitumor immunity.

The microbiota is a critical component of the digestive system. It encompasses bacteria, fungi, viruses, and archaea. It maintains mucosal barrier integrity and regulates local immune homeostasis. A healthy microbiota strengthens the mucosal barrier by producing more mucus, accelerating epithelial healing, and enhancing immune resilience. Hypoxia, on the other hand, disrupts this equilibrium. This disruption leads to dysbiosis, in which potentially pathogenic taxa such as Proteobacteria and Bacteroides proliferate, while beneficial commensals, such as *Lactobacillus* and *Bifidobacterium*, decline (Chen L. R. et al., 2025; Wang M. et al., 2025). This dysbiosis compromises barrier integrity, perpetuates chronic inflammation, and facilitates colonization by pathogens such as *Helicobacter pylori*. Pathogen-derived factors like CagA and VacA worsen hypoxia and inflammation, leading to a self-perpetuating cycle of mucosal damage (Chivu et al., 2025; Fiorani et al., 2023). Hypoxia alters the profile of microbial metabolites, such as short-chain fatty acids and bile acids, which directly affect host signaling pathways. This alters epithelial function, immune cell recruitment, and metabolic balance (Zhang et al., 2025; Zhang Z. et al., 2024). There is a bidirectional relationship between hypoxia and the stomach microbiota. Together, they determine mucosal health and disease susceptibility.

High-throughput sequencing and metabolomics have significantly advanced our understanding of the “microbiota-metabolism axis” in hypoxic environments. Integrated multi-omics studies using transcriptomic and proteomic data have identified genetic markers in gastric cancer associated with hypoxia and the immune system, which are crucial for predicting disease progression. They have elucidated the interactions among hypoxia, immunological regulation, and microbial dynamics (Liu et al., 2020; Pei et al., 2021; Wang X. et al., 2025). Metabolomic studies demonstrate that hypoxia alters the concentrations of microbial metabolites, including butyrate, propionate, and secondary bile acids. These alterations weaken the epithelial barrier and trigger inflammatory responses. In studies utilizing gastric cancer models, metabolic changes induced by hypoxic conditions have been demonstrated to facilitate tumor proliferation, enhance resistance to chemotherapy, and evade immune detection (Wang et al., 2022; Xu et al., 2024). Consequently, therapeutic approaches targeting this axis—such as probiotics, fecal microbiota transplantation, and herbal formulations—are emerging as promising strategies to restore microbial equilibrium, facilitate mucosal healing, and enhance treatment efficacy (Cao et al., 2022; Xu et al., 2025). These findings underscore the importance of understanding the molecular architecture of the “hypoxia-microbiota-metabolite” network for translating research into practical applications.

**Figure 1:**
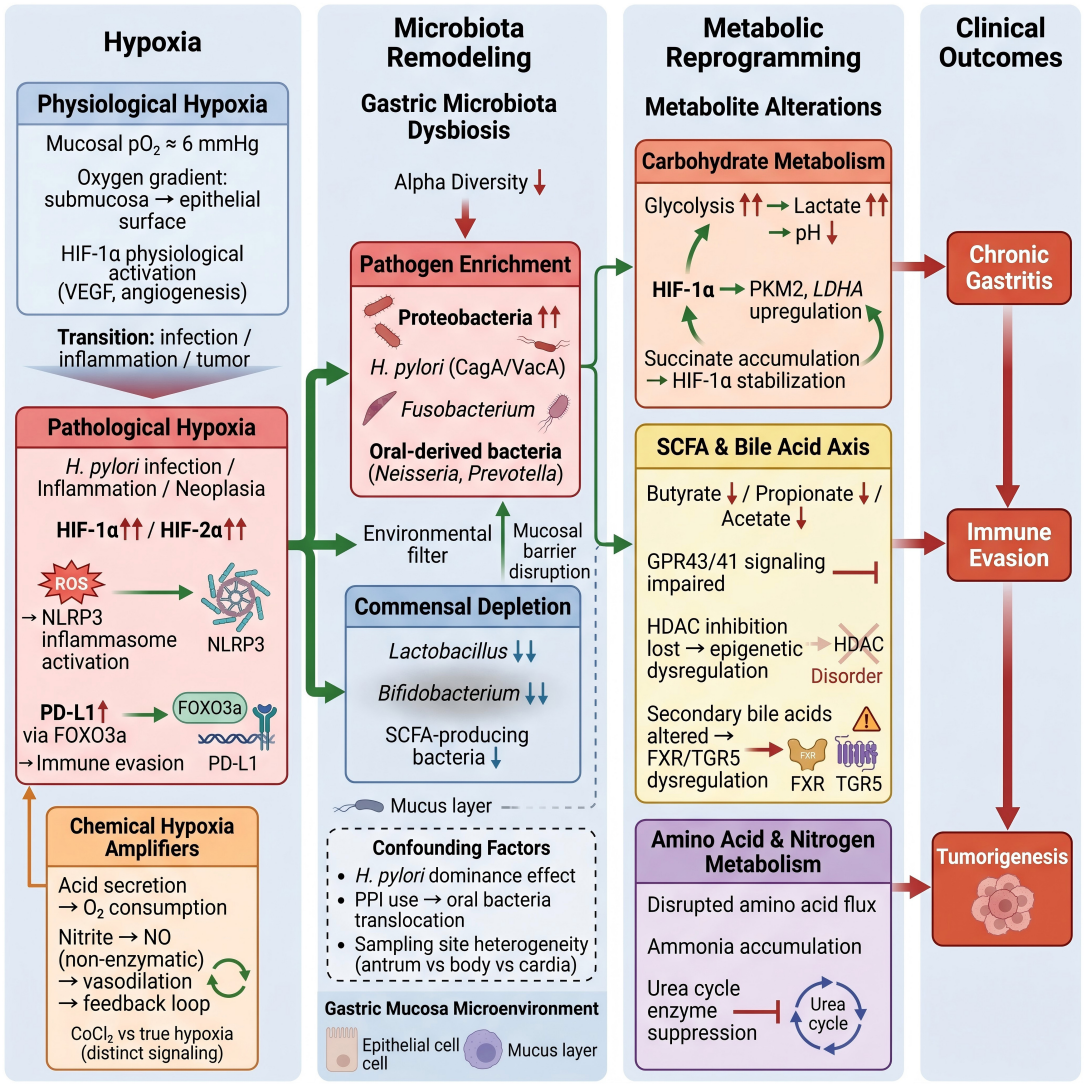
Integrated Mechanisms of the Gastric Microenvironment: Hypoxia, Microbiota Dysbiosis, Metabolic Reprogramming, and Immune Evasion.

Developing novel therapies for gastric disorders requires a comprehensive understanding of how hypoxia influences the gastric microbiota and its metabolic byproducts. Hypoxia causes dysbiosis and metabolic dysfunction, which, in turn, lead to long-term inflammation, barrier breakdown, and immune dysregulation. This accelerates the progression of gastritis, peptic ulcers, and gastric cancer (Zhou et al., 2023; Chen L. R. et al., 2025; Chivu et al., 2025). Hypoxia and microbial metabolites interact to alter the function of epithelial and immune cells, thereby influencing disease progression rates and therapeutic efficacy (Xu et al., 2024; Koga, 2022). Recent prognostic models that examine gene expression associated with low oxygen levels, immune markers, and microbial profiles have facilitated the identification of at-risk individuals and may eventually guide more tailored therapeutic approaches, although these models remain preliminary and require prospective validation, for gastric cancer (Liu et al., 2020; Chen et al., 2020). These findings support therapeutic strategies targeting the hypoxia-microbiota-metabolism axis, including HIF inhibition, microbiota restoration, or metabolite-guided immunomodulation to restore mucosal and metabolic homeostasis. Further investigation of this axis is warranted to explore the potential for more individualized diagnostic and therapeutic approaches, though current evidence does not yet support definitive clinical application for gastric cancer. An overview of the integrated mechanisms underlying this axis is illustrated in Figure 1

### 1.1 Methods

A systematic literature search was conducted in accordance with the PRISMA 2020 guidelines. Three electronic databases—PubMed, Web of Science Core Collection, and Scopus—were searched from inception through March 2025 to identify studies examining the relationship between hypoxia and the gastric microbiota-metabolism axis. The search strategy combined the following key terms using Boolean operators: (“hypoxia” OR “hypoxic” OR “low oxygen” OR “oxygen deprivation” OR “hypoxia-inducible factor” OR “HIF”) AND (“gastric microbiota” OR “stomach microbiota” OR “gastric microecology” OR “gastric flora” OR “gastric microbiome”) AND (“metabolism” OR “metabolomics” OR “metabolic reprogramming” OR “metabolite” OR “short-chain fatty acid” OR “bile acid”). The search was restricted to English-language, peer-reviewed articles published between January 2000 and March 2025.

Inclusion criteria were: (1) original research or comprehensive reviews addressing the effects of hypoxia on gastric microbial composition, metabolic output, or both; (2) studies employing high-throughput sequencing, metabolomics, or integrated multi-omics approaches; and (3) investigations of gastric diseases (gastritis, peptic ulcer disease, or gastric cancer) in which hypoxia was discussed as a contributing factor. Exclusion criteria were: conference abstracts without full-text availability, non-English publications, and studies focusing exclusively on extra-gastric microbiota without any discussion of relevance to the gastric niche.

The initial search yielded 847 records. After removal of duplicates (*n* = 312), 535 unique records underwent title and abstract screening. Of these, 78 articles were selected for full-text review, and 51 met the inclusion criteria. Additionally, reference lists of included articles and relevant review papers were manually screened to identify additional studies, yielding 16 supplementary records. In total, 67 studies were included in this review.

## 2 Body

### 2.1 Effects of hypoxia on gastric microbial community composition

#### 2.1.1 Mechanisms of physiological hypoxia in the stomach

The physiological hypoxia in the stomach mucosa arises from its distinctive hemodynamic properties and increased metabolic requirements. The mucosal microvasculature generates a steep oxygen gradient from the well-oxygenated submucosa to the epithelial surface. The mucosal surface exhibits consistently low oxygen tension due to limited perfusion and the high metabolic demand of acid-secreting parietal cells. This oxygen gradient is critical for maintaining cellular differentiation and preserving tissue integrity. However, this gradient also renders the mucosa susceptible to pathological hypoxia under conditions of vascular or metabolic stress.

The stomach produces gastric acid to help digest food and maintain stable oxygen levels. Rapid acid secretion consumes oxygen, thereby reducing local partial pressure. Other chemical processes occur in the acidic environment produced, including the formation of ascorbic acid, which catalyzes the conversion of nitrite to nitric oxide (NO) without the need for enzymes. Gastric microbiota supply nitrite, a necessary substrate for this non-enzymatic pathway. This non-enzymatic NO production becomes particularly important under hypoxic conditions, when the conventional L-arginine-NOS pathway is compromised. Subsequently, NO induces vasodilation, modulating mucosal blood flow and oxygen delivery. This creates a strong feedback loop that maintains a steady physiological hypoxic microenvironment.

Hypoxia-inducible factors (HIFs) mediate the principal adaptive response, with HIF-1α serving as the central regulator. When HIF-1α is activated in physiological hypoxia, it protects the mucosa by increasing the expression of genes involved in angiogenesis (such as VEGF), metabolism, and cell survival. It also alters adhesion molecules, such as CEACAM6 (Poirah et al., 2024), increasing susceptibility to *H. pylori* infection and other pathologies. In summary, gastric physiological hypoxia represents a distinctive feature that derives from a mix of hemodynamic, metabolic, chemical, and microbiological factors. These variables work together to establish the important oxygen gradient and initiate adaptive responses, primarily through HIF signaling. Further investigation of this complex regulatory system is warranted to elucidate how the gastric mucosa maintains homeostasis and what occurs when it becomes diseased, particularly under conditions of infection or ischemia (Du et al., 2023; Wang X. et al., 2025).

In the healthy stomach, the mucosal surface is characterized by a steep oxygen gradient from the well-oxygenated submucosal capillary bed to the epithelial layer, which exists in a state of physiological hypoxia, although direct measurements of human gastric mucosal pO_2_ remain limited in the literature, animal studies consistently demonstrate that the gastric epithelium operates under markedly low oxygen tensions. In a well-controlled study examining transmural oxygen gradients across the gastrointestinal tract in rats, the gastric mucosal pO_2_ was measured at 6 ± 1 mmHg under normoxic conditions, whereas the colonic mucosa was higher at 10 ± 2 mmHg (Walker et al., 2021). This steep gradient arises from a combination of unique microvascular anatomy and high metabolic demands of acid-secreting parietal cells. Consistent with this observation, the healthy colonic mucosa is widely reported to exist at a pO_2_ of less than 10 mmHg. These findings establish that the gastric and colonic mucosae are constitutively hypoxic tissues that are fundamentally distinct from highly oxygenated compartments such as the lung alveolus (100–110 mmHg).

#### 2.1.2 Microbial community shifts under declining oxygen tensions

Hypoxia is a powerful environmental filter that has a lasting impact on microbial communities. Its most fundamental effect is to disrupt the delicate balance between specialized anaerobic bacteria and facultative anaerobes, thereby triggering a series of reactions that lead to dysbiosis. *H. pylori* and other anaerobic bacteria may adapt their energy metabolism to survive in hypoxic environments (such as the stomach). In contrast, oxygen-sensitive beneficial anaerobic bacteria, such as *Bifidobacterium* and *Lactobacillus*, may face increased competition for substantial nutrients (Han et al., 2022; Guo et al., 2024). Hypoxia drives community restructuring, leading to a continuous decline in alpha diversity (the number of species in a community) and a change in β diversity (the difference in species composition between communities). Collectively, these shifts indicate ecosystem progression toward a composition detrimental to host health. A summary of key studies on the gastric microbiota under hypoxic or hypoxia-related conditions is presented in Table 1.

**Table 1:** Summary of key studies on the gastric microbiota under hypoxic or hypoxia-related conditions.

References	Sample type	Sample size	Method	Disease context	Key findings	*H. pylori* controlled?
(Sharma et al. 2025)	Gastric mucosal biopsy	*H. pylori*+ GERD/duodenal ulcer/gastritis patients	16S rRNA sequencing	Gastritis, GERD, duodenal ulcer	Significant alteration of gastric microbiota in *H. pylori*-infected individuals; enrichment of *Streptococcus infantis* and *Bacteroides* spp.	Yes (*H. pylori*+ cohort)
(Zheng et al. 2022)	Gastric mucosal biopsy	Children with duodenal ulcer	16S rRNA sequencing	Duodenal ulcer	*H. pylori* infection alters gastric microbiota composition; reduced microbial diversity in infected children	Yes
(Gunathilake et al. 2019)	Gastric mucosal biopsy	268 GC patients, 288 controls	16S rRNA sequencing	Gastric cancer	*H. pylori* relative abundance positively associated with GC risk (OR = 1.86); association attenuated after adjustment	Partially (adjusted in regression)
(Cervantes et al. 2021)	Oral, gastric, duodenal samples	Upper GI symptom patients	16S rRNA sequencing	Upper GI symptoms	Oral-derived taxa (*Neisseria, Prevotella*) enriched in gastric samples of patients with upper GI pathology	Not specified
(Zhou et al. 2023)	Gastric tissue	GC patients	Metagenomic sequencing	Gastric cancer	Gastric cancer microbiome enriched in oral-derived commensals (*Neisseria, Prevotella, Fusobacterium, Peptostreptococcus*); depletion of *Lactobacillus* and *Bifidobacterium*	Not specified
(Lv et al. 2025)	Gastric cancer tumor tissue	GC patients	16S rRNA + culture	Gastric cancer	Live bacteria identified within gastric cancer tumor tissue; hypoxic TME may support bacterial survival	Not specified
(Oosterlinck et al. 2023)	Gastric mucosal biopsy	GC patients	16S rRNA + metagenomics	Gastric cancer	Mucin-microbiome signatures shape the tumor microenvironment; mucin degradation-associated taxa enriched	Not specified
(Wang et al. 2023)	Gastric mucosal biopsy	GC patients	16S rRNA sequencing	Gastric cancer	*H. pylori* infection reduces bacterial diversity; spirochetes enriched; *Mycobacterium* and *Lactobacillus* depleted	Yes
(Chen Z. et al. 2025)	Gastric tissue	GC patients	Metagenomic sequencing	Gastric cancer	Comprehensive profiling of gastric microbiota in carcinogenesis; functional capacity altered under hypoxic conditions	Partially
(He et al. 2024)	Gastric tissue	Post-gastrectomy patients	Metabolomics + 16S rRNA	Postoperative insulin resistance	Postoperative insulin resistance associated with altered gastric microbiota and metabolite profiles	Not applicable
(Shi et al. 2019)	Gastric mucosal biopsy	GERD patients on PPIs	16S rRNA sequencing	GERD (PPI effects)	PPI use elevates oral-derived taxa (*Planococcaceae, Oxalobacteraceae, Sphingomonadaceae*) in gastric mucosa	No
(Chen Z. et al. 2025)	Gastrointestinal mucosa	GI injury model	16S rRNA + metabolomics	Hypoxia-induced GI injury	Hypoxia disrupts mucus system and intragastric microecology; Proteobacteria expansion, beneficial taxa depletion	N/A (animal model)
(Bai et al. 2022)	Fecal/gut samples	Rats under high-altitude hypoxia	16S rRNA sequencing	High-altitude hypoxia	Hypoxia promotes facultative anaerobes, suppresses obligate anaerobes; metabolic and immune disturbances	N/A (animal model)
(Zhou et al. 2021)	Intestinal tissue	Mice under intermittent hypoxia	Transcriptomics + 16S rRNA	Intermittent hypoxia	Microbial colonization significantly influenced cardiac transcriptional patterns under intermittent hypoxia	N/A (animal model)

In summary, hypoxia is a major factor in the compositional shift of microbial communities. It favors facultative anaerobes while suppressing obligate anaerobes. Environmental shifts displace resident microbial populations, progressively diminishing community diversity and functional capacity. This decline has a significant impact on the host's health, the risk of disease, and the stability of the ecosystem. Therefore, it is crucial to understand, at the molecular level, how bacteria respond to hypoxic conditions to inform the development of therapeutics for dysbiosis-related diseases.

#### 2.1.3 Metabolite alterations under hypoxic conditions

In the absence of sufficient oxygen, gastric bacteria alter their metabolic pathways, leading to significant changes in the molecular composition of the stomach. In a hypoxic environment, fermentative bacteria dominate, altering their energy utilization mechanisms. Due to hypoxia, lactic acid accumulates in tissues as a result of anaerobic glycolysis. This occurs when the hypoxia-inducible factor (HIF) induces glycolysis and alters the composition of the microbial community. An increase in lactic acid levels lowers the pH of the surrounding environment. This acidification compromises the epithelial barrier and disrupts microbial homeostasis. Hypoxia significantly affects the production of short-chain fatty acids (SCFAs) by microorganisms, including acetic, propionic, and butyric acids. The disappearance of beneficial bacteria (such as *Lactobacillus* and *Methanobrevibacter*) usually reduces the total SCFA level, thereby impairing mucosal immune function and barrier integrity. However, new data show that changes in the short-chain fatty acid (SCFA) pattern caused by hypoxia reveal a mismatch between microbial metabolic flexibility and the host's physiological needs.

The major metabolite alterations in the hypoxic gastric microenvironment are summarized in Table 2. Hypoxia alters the network responsible for amino acid and nitrogen catabolism, significantly affecting the balance of the gastric mucosa. The content of tryptophan and glutamine fluctuated significantly. Tryptophan metabolites produced by the kynurenine pathway and microbial indole synthesis play a key role in regulating the immune response and barrier function (Qi et al., 2023; Zeng et al., 2025). The other two amino acids that help tissue repair are asparagine and ornithine. They also alter the function of immune cells (especially macrophages) and the stomach (Díaz-Bulnes et al., 2019). Hypoxia will accelerate nitrogen fixation and the removal of bacterial enzymes. This contradicts the nitrogen cycle in the stomach. When this happens, nitrogen-containing compounds such as ammonia accumulate, increasing the pH in the surrounding area and altering the function of epithelial cells. In models of hypoxia-induced cognitive impairment, changes in the tryptophan system are associated with alterations in nitrogen utilization, suggesting that these changes affect the whole organism (Qi et al., 2023). The accumulation of nitrogen metabolites is associated with a complex that is harmful to organisms. These fundamental metabolic disturbances compromise immune function, hinder tissue repair, and destabilize the gastric environment. Metabolomics screening of patients with peptic ulcer disease (PUD) found that these microecologies and microbiomes with hypoxia and flora disorders can exacerbate inflammation, as evidenced by increased IL-6 and NF-κB levels in a gastrointestinal injury model (Tian et al., 2024; Zhong et al., 2024). This indicates a relationship between the metabolic and inflammatory processes.

**Table 2:** Major metabolite alterations in the hypoxic gastric microenvironment.

Metabolite category	Specific metabolites	Direction of change under hypoxia	Proposed mechanism	Evidence type	Key limitations
Short-chain fatty acids (SCFAs)	Butyrate, propionate, acetate	↓ Decreased	Depletion of SCFA-producing commensals (*Lactobacillus, Bifidobacterium, C. butyricum*) reduces HDAC inhibition, anti-tumor immunity, and barrier integrity (Yang and Cong, 2021; Cao et al., 2022)	Human + animal studies	Most evidence from intestinal/gut models; direct gastric SCFA measurements limited
Lactic acid	Lactate	↑ Increased	HIF-1α-driven glycolytic shift (Warburg effect) + bacterial fermentation; acidifies microenvironment, promotes angiogenesis via HIF-1α/VEGF, suppresses CTL and NK cell function (Aird et al., 2022; Wang et al., 2022)	Human (GC) + animal models	Difficult to distinguish host-derived vs. bacteria-derived lactate *in vivo*
Succinate	Succinate	↑ Increased	Intracellular accumulation in macrophages stabilizes HIF-1α via PHD inhibition; creates positive feedback loop amplifying glycolysis and inflammation (Jiang et al., 2021; Li et al., 2024)	Primarily animal/cell models	Gastric-specific succinate data scarce; mostly from Brucella infection models
Bile acids	Secondary bile acids, taurine-conjugated bile acids	Altered (direction variable)	Dysregulated bile acid metabolism alters FXR/TGR5 signaling; affects drug transport, inflammation, and energy balance (Zhang et al., 2025; Lau et al., 2021; Münzker et al., 2022; Hanna et al., 2025)	Human (post-bariatric surgery, GC)	Gastric bile acid data primarily from post-surgical contexts; normal gastric bile acid concentrations poorly characterized
Tryptophan catabolites	Indole, kynurenine, AhR ligands	Altered (direction variable)	Kynurenine pathway and microbial indole synthesis regulate immune responses and barrier function; AhR activation promotes MDSC expansion (Cervantes et al., 2021; Chen Z. et al., 2025; Singhal and Shah, 2020; Lau et al., 2021)	Human + animal models	Most tryptophan metabolism studies from intestinal, not gastric, contexts
Nitrosamines	N-nitroso compounds	↑ Increased	Nitrate-reducing bacteria generate nitrosamines under acidic, hypoxic conditions; DNA damage via O6-methylguanine adducts (Poirah et al., 2024; Savitri et al., 2025)	Primarily mechanistic/experimental	Direct quantification of gastric nitrosamines from microbial sources *in vivo* is limited
Amino acids	Glutamine, asparagine, ornithine	Fluctuating (context-dependent)	Glutamine and asparagine support tissue repair; ornithine alters macrophage function; nitrogen catabolism disrupted (Liu et al., 2024; Lv et al., 2025)	Animal + metabolomics studies	Gastric-specific amino acid dynamics under hypoxia poorly characterized
Adenosine	Adenosine	↑ Increased	Hypoxic conditions elevate extracellular adenosine; suppresses T cell proliferation and cytokine production via A2A receptor (Song et al., 2025)	Primarily tumor immunology models	Direct measurement in gastric mucosal microenvironment lacking
Polyamines	Spermine, spermidine	Altered	Amino acid-derived polyamines affect cell proliferation and mucosal repair; bile acid degradation may alter polyamine availability (Münzker et al., 2022)	Indirect (metabolomics)	Mechanistic link to hypoxia in gastric context remains speculative

In summary, hypoxia has a significant impact on the gastric microbiota-metabolic axis through multiple mechanisms. The main changes in metabolism include enhanced glycolysis leading to the formation of lactic acid, shifts in the spectrum of short-chain fatty acids produced by fermentation communities, and a complete reorganization of amino acid and nitrogen utilization. These fundamental metabolic disturbances compromise immune function, hinder tissue repair, and destabilize the gastric environment. These disturbances exacerbate the severity of gastric diseases. We need to better understand how hypoxia affects metabolism to elucidate the functional mechanisms of stomach diseases and identify broadly applicable therapeutic strategies.

#### 2.1.4 Metabolic reprogramming under hypoxic conditions

When oxygen is insufficient, metabolic reprogramming is an important way for host cells and microbial communities to survive. This adaptation entails a shift in energy metabolism to anaerobic pathways, primarily glycolysis and fermentation. The hypoxic-inducing factor HIF-1α and other related factors control this transformation. They suppress mitochondrial oxidative phosphorylation and upregulate genes involved in glucose metabolism. Due to this metabolic change, cells will quickly absorb glucose and produce excess lactic acid, making the tissue highly acidic (Zhong et al., 2024). *H. pylori* and *Candida albicans* are two microorganisms that reside in the stomach. These organisms activate fermentation pathways and glycolytic enzymes, conferring a proliferative advantage in hypoxic niches (Burgain et al., 2020; Qi et al., 2023).

More importantly, short-chain fatty acids, succinic acid, and inosine are by-products of microbial metabolism. They are important signal molecules that directly affect the host's physiological function. For example, inositol from *Lactobacillus* Johnson alters B cell growth via the ERK-HIF-1α pathway. However, when macrophages are infected with Brucella, intracellular succinic acid accumulates, thereby stabilizing hypoxia-inducible factor-1α. This will increase the flow of glycolysis and the production of pro-inflammatory cytokines, thus helping the human body fight infection (Gomes et al., 2021; Gao et al., 2025). These pathways show the complex interactions between different biological worlds, in which microorganisms directly alter the functioning of the human immune system and metabolism.

Accumulating evidence indicates a bidirectional biochemical interplay. The metabolites produced by the host can alter the microbial composition within the host, but metabolic reprogramming directly alters the metabolism of host cells. This dynamic relationship alters the connection between the microbiome and metabolism, affecting conditions such as gastric cancer. Notably, the impact of this metabolic change extends beyond fermentation and glycolysis. It has also significantly remodeled the biochemical networks of nitrogen metabolism, amino acids, and nucleotides. Hypoxia significantly impacts both the microbiome and gastric metabolism. This occurs because hypoxia alters key energy pathways, prompting the use of bacterial metabolites to maintain stable energy levels. To understand the mechanisms underlying hypoxia-related diseases and develop new treatments, it is necessary to characterize the interactions among all relevant components fully.

On the other hand, changes in metabolites disrupt the balance between Treg and Th17 cells, leading to pro-inflammatory phenotypes that worsen mucosal injury (Wang et al., 2024; Yang and Cong, 2021). These metabolites can also affect immune system proteins after translation. These metabolites can also modulate post-translational modifications of immune proteins, influencing immune evasion or antitumor responses.

### 2.2 Hypoxia modifies the gastric microbiota-metabolism axis and its association with gastric disorders

#### 2.2.1 Changes in the microbiota-metabolism axis in people with gastritis and stomach ulcers

Gastric mucosal hypoxia plays a pivotal role in the development of gastritis and gastric ulcers by disrupting the microbiota-metabolism axis. This facilitates colonization and virulence enhancement of pathogenic bacteria, notably *H. pylori*. *H. pylori* infection, which is the main cause of chronic gastritis and peptic ulcers, significantly alters the bacteria that live in the stomach. 16S rRNA sequencing shows that the gastric microbiota of *H. pylori*-infected individuals undergo significant alterations. Several bacteria, such as *Streptococcus infantis* and *Bacteroides* spp. (Yoon et al., 2023; Sharma et al., 2025), can cause ulcers and gastritis. *H. pylori* not only maintains a microaerophilic environment conducive to its own growth but also inhibits the growth of other bacteria. These observed differences indicate a reduction in microbial abundance and diversity. This dysbiosis destabilizes microbial co-expression networks, predisposing to broader mucosal barrier dysfunction. The study found 66 molecules with altered content and 12 metabolic pathways with significant changes. This clearly shows a systematic relationship between microbial dysbiosis and the pathophysiology of mucosal damage.

Bacterial metabolic byproducts can damage the gastric mucosa under hypoxic conditions, as they disrupt important biological processes, such as energy metabolism (Zheng et al., 2022). Fructose-1,6-diphosphatase III activity is low in patients with gastric ulcers and gastritis. This suggests that glycogenesis may be impaired, leading to insufficient energy for epithelial cells and preventing the repair process from proceeding normally (Yoon et al., 2023). In this study, hypoxia triggered a disease cycle: *H. pylori* infection leads to dysbiosis of the flora and metabolic dysfunction, thereby forming a vicious circle of “metabolic damage—mucosal damage—persistent inflammation.” A deeper understanding of the mechanisms underlying gastritis and gastric ulcer formation will enhance the appreciation of hypoxia's impact on bacterial and metabolic processes, facilitating the identification of novel strategies to restore microbial balance and mucosal barrier function.

#### 2.2.2 Hypoxia-driven microbiota-metabolism axis remodeling in gastric carcinogenesis

The tumor microenvironment of gastric cancer is characterized by profound and sustained hypoxia, which fundamentally distinguishes it from the physiological hypoxia of the normal gastric mucosa. Rapid tumor cell proliferation outpaces angiogenesis, resulting in structurally abnormal vasculature with erratic blood flow and oxygen delivery. This pathological hypoxia stabilizes HIF-1α and HIF-2α constitutively, transforming these transcription factors from adaptive regulators into oncogenic drivers. HIF-1α reprograms cellular metabolism toward aerobic glycolysis—the Warburg effect—by upregulating glucose transporters (GLUT1) and glycolytic enzymes (HK2, PKM2, and LDHA), thereby providing proliferative advantage even in oxygen-depleted conditions (Wang et al., 2022). Beyond metabolic rewiring, HIF-1α promotes epithelial-mesenchymal transition (EMT) through transcriptional activation of SNAIL, TWIST, and ZEB1, enhancing invasive potential and metastatic capacity (Wang M. et al., 2025; Zhang Z. et al., 2024). HIF-2α, while sharing overlapping targets with HIF-1α, exerts distinct oncogenic functions including the regulation of c-MYC and cyclin D1, further driving cell cycle progression in gastric cancer cells. These HIF-mediated programs establish a self-reinforcing oncogenic circuitry that not only sustains tumor growth but also actively reshapes the surrounding microbial and metabolic landscape.

Hypoxia-driven dysbiosis in the gastric tumor microenvironment exhibits a characteristic microbial signature distinct from that of non-neoplastic gastric conditions. Metagenomic and 16S rRNA sequencing studies have consistently demonstrated that the gastric cancer microbiome is enriched in oral-derived commensals—particularly *Neisseria, Prevotella, Fusobacterium*, and *Peptostreptococcus*—while obligate anaerobes associated with mucosal health, such as *Lactobacillus* and *Bifidobacterium*, are significantly depleted (Zhou et al., 2023; Vanderhaeghen et al., 2020; Aird et al., 2022). This oralization of the gastric microbiome may reflect both the elevated intragastric pH resulting from chronic atrophic gastritis and the hypoxia-mediated disruption of the mucosal barrier, which permits translocation of oropharyngeal bacteria. *Fusobacterium nucleatum*, in particular, has emerged as a potent oncomicrobe in gastric cancer: its FadA adhesin binds E-cadherin on gastric epithelial cells, activating β-catenin signaling and promoting cell proliferation, while its lipopolysaccharide (LPS) engages TLR4/NF-κB signaling to sustain a pro-inflammatory milieu (Chivu et al., 2025). In a large-scale case-control study involving 268 gastric cancer patients and 288 controls, Gunathilake et al. (2019) demonstrated that the relative abundance of *H. pylori* was significantly positively associated with gastric cancer risk (OR = 1.86, 95% CI: 1.17–2.97), although this association was substantially modified after adjusting for *H. pylori* status, underscoring the confounding role of this dominant colonizer. These microbial shifts are not merely bystander effects; specific taxa actively contribute to carcinogenesis through defined molecular mechanisms, creating a dysbiotic niche that reinforces the hypoxic tumor microenvironment.

Microbial metabolites in the hypoxic gastric microenvironment exert dual—and often opposing—influences on carcinogenesis. On the pro-tumorigenic side, nitrosamines generated by nitrate-reducing bacteria (Liu et al., 2023) under acidic, hypoxic conditions directly damage DNA through the formation of O6-methylguanine and other carcinogenic adducts, initiating mutagenesis in gastric epithelial cells. Elevated lactic acid, a product of both host glycolysis and bacterial fermentation, acidifies the microenvironment and promotes angiogenesis via HIF-1α-dependent VEGF upregulation, while simultaneously suppressing cytotoxic T lymphocyte and NK cell function, thereby facilitating immune evasion. Succinate, which accumulates intracellularly in macrophages infected by intracellular bacteria, stabilizes HIF-1α through competitive inhibition of prolyl hydroxylases, creating a positive feedback loop that amplifies both glycolysis and inflammatory cytokine production. Conversely, short-chain fatty acids—particularly butyrate—exert anti-tumor effects by inhibiting histone deacetylases (HDACs), thereby reactivating tumor suppressor genes and promoting apoptosis in gastric cancer cells. Butyrate also enhances the integrity of the epithelial barrier and modulates immune cell function toward an anti-tumor phenotype. However, the depletion of SCFA-producing commensals (*Lactobacillus, Bifidobacterium*, and *Clostridium butyricum*) in the hypoxic gastric niche tilts this balance decisively toward tumorigenesis (Cao et al., 2022). The eventual equilibrium between pro- and anti-tumorigenic metabolites is thus dictated by the composition of the microbiome and its metabolic output under oxygen-limited conditions.

A critical consequence of hypoxia-driven microbiota-metabolism axis remodeling is the establishment of an immunosuppressive tumor microenvironment. HIF-1α upregulates PD-L1 expression on gastric cancer cells through both direct transcriptional activation and the FOXO3a signaling pathway, enabling tumor cells to engage PD-1 on cytotoxic T lymphocytes and thereby escape immune surveillance (Xiang et al., 2025). This immune evasion is compounded by metabolite-mediated immune modulation: the depletion of SCFAs impairs regulatory T cell (Treg) function while promoting Th17 polarization, and elevated lactate and adenosine in the hypoxic niche further suppress T cell proliferation and cytokine production (Qi et al., 2023). Concurrently, hypoxia induces ferroptosis—a regulated form of iron-dependent cell death driven by lipid peroxidation—through the coordinated upregulation of HIF-1α and HIF-2α. While ferroptosis can theoretically suppress tumor growth by eliminating damaged cells, its dysregulation in the hypoxic gastric microenvironment paradoxically promotes carcinogenesis: ferroptotic cell debris releases damage-associated molecular patterns (DAMPs) that recruit and polarize macrophages toward an M2 tumor-promoting phenotype, and the iron overload generated by ferroptotic cell death catalyzes further ROS production and DNA damage (Xu et al., 2024). Integrated multi-omics analyses have revealed that ferroptosis-related genes in the hypoxic gastric cancer microenvironment delineate distinct immune landscapes, offering potential—though still preliminary—for patient stratification and the future development of more tailored therapeutic strategies (Xu et al., 2024).

Hypoxia-driven remodeling of the microbiota-metabolism axis also underpins chemotherapy resistance in gastric cancer. HIF-1α upregulates multidrug resistance protein 1 (MDR1/ABCB1) and vascular endothelial growth factor (VEGF), collectively reducing intracellular drug accumulation and promoting a pro-survival vascular niche (Wang et al., 2022). Metabolically, the glycolytic shift induced by hypoxia depletes ATP required for drug efflux pump function paradoxically while simultaneously activating alternative survival pathways, including the PI3K/AKT/mTOR axis, that render cancer cells less susceptible to apoptosis induced by 5-fluorouracil and cisplatin. Microbial metabolites further modulate chemoresistance: dysregulated bile acid metabolism alters FXR/TGR5 signaling, which has been implicated in drug transport and metabolism, while tryptophan catabolites acting through the aryl hydrocarbon receptor (AhR) promote the expansion of immunosuppressive myeloid-derived suppressor cells (MDSCs) that blunt the efficacy of both chemotherapy and immunotherapy (Zhang et al., 2025; Gutiérrez-Repiso et al., 2021). Notably, metabolomic profiling has revealed that changes in the ratio of *Klebsiella* to *Bacteroides* and alterations in amino acid catabolism in gastric cancer patients correlate with neoadjuvant chemotherapy response, suggesting that microbiome-metabolite signatures could serve as predictive biomarkers for treatment stratification (He et al., 2024; Zhang P. et al., 2024).

Importantly, gastric cancer itself amplifies the hypoxic conditions that drove its initiation, creating a self-reinforcing vicious cycle. Tumor growth increases oxygen consumption, aberrant neovascularization fails to meet metabolic demand, and tumor-associated macrophages and myeloid cells further consume oxygen in the production of ROS and inflammatory mediators. This deepening hypoxia perpetuates HIF stabilization, which in turn sustains the dysbiotic microenvironment by further suppressing beneficial commensals and promoting pathogen enrichment. The resulting metabolic milieu—characterized by lactate accumulation, SCFA depletion, and nitrosamine generation—not only accelerates tumor progression but also primes the surrounding tissue for additional neoplastic transformation, potentially explaining the field cancerization effect observed in gastric carcinogenesis. Interrupting this vicious cycle at any node—whether by HIF inhibition, targeted microbiota restoration, or metabolic pathway modulation—represents a rational therapeutic strategy, although the optimal combination and timing of such interventions remain to be determined through longitudinal interventional studies.

#### 2.2.3 Additional research on gastric disorders

Functional gastrointestinal disorders (FGIDs), such as functional dyspepsia, are challenging to manage due to their multifactorial etiology. Hypoxia is a key pathophysiological link that has recently been identified. Under conditions of oxygen deprivation, hypoxia-inducible factors (HIFs) are activated, which then control the production of inflammatory mediators, modulate cellular energy metabolism and innate immune responses, damage the gastric mucosa, and promote microbial dysbiosis (Chen L. R. et al., 2025). Individuals with FGIDs exhibit alterations in their gastrointestinal microbiota. These changes, which include increased anaerobic metabolism and a wider range of species, were probably caused by localized hypoxic microenvironments. These microbial alterations exacerbate mucosal inflammation and impair gastric motility.

This initiates a vicious cycle wherein metabolic dysfunction, dysbiosis, and hypoxia mutually exacerbate one another. Preclinical investigations show that hypoxia induces ferroptosis, a regulated form of cell death, by elevating HIF-1α and HIF-2α levels, which directly harm epithelial cells.

By restoring oxygen balance, altering bacterial interactions, and controlling metabolic pathways, scientists can develop novel medications to treat FGID symptoms. The development of probiotics and phytomedicines targeting the microbiota must account for the prevailing hypoxic environment. For instance, agarwood essential oil has shown promise as an additional therapy that protects against damage induced by low oxygen levels by halting lipid peroxidation and facilitating recovery from microbial-metabolic disorders (Wang X. et al., 2025).

Researchers are continually exploring innovative techniques to treat hypoxic conditions in the stomach directly. For example, exosome-like nanoparticles derived from Robinia pseudoacacia flowers (RFELN) have been shown to protect gastric mucosal cells from ferroptosis by mitigating HIF-1α/2α-induced oxidative stress, while concurrently promoting microbial and metabolic repair. *H. pylori* infection models underscore the systemic relevance of these therapeutic approaches.

In the future, advanced therapeutics that simultaneously modulate the microbiota and directly target hypoxia (e.g., antioxidants or HIF inhibitors) to effectively treat hypoxia-associated gastric disorders. The primary objective of treatment should be to restore oxygen levels to normal, manage microbial communities, and address metabolic imbalances. Such an approach aims to improve clinical outcomes for patients with FGIDs and other hypoxia-associated gastrointestinal disorders.

### 2.3 Methods of research and progress in technology

#### 2.3.1 High-throughput sequencing approaches for gastric microbiota characterization

High-throughput sequencing has altered the way we study the gastric Microbiota by overcoming the challenges posed by the stomach's acidity. Currently, metagenomic sequencing and 16S rRNA gene sequencing are the two most widely used methods for such investigations. Each method has its own advantages and limitations. This approach identifies bacterial taxa by targeting conserved genomic regions shared across diverse species. This cost-effective method facilitates elucidation of how *H. pylori* infection alters microorganisms and increases the risk of gastric cancer (Cervantes et al., 2021; Chen Z. et al., 2025). Although valuable for taxonomic surveys, this method is typically limited to genus-level resolution and provides no direct functional information. Metagenomic sequencing, on the other hand, analyzes the genomes of entire bacterial communities. It can simultaneously detect diverse kingdoms, including bacteria, fungi, viruses, and archaea. It also elucidates functional capacity and metabolic potential. This comprehensive approach has significantly advanced our understanding of how microorganisms influence gastric pathophysiology (Liu et al., 2024; Lv et al., 2025).

Before using genetic data to obtain useful biological information, extensive bioinformatics research is required. When processing 16S data, the usual practice is to assess its quality, remove chimeric sequences, and organize it into operational taxonomic units (OTUs) or amplified subsequence variants (ASVs) to improve clarity. After that, use reference databases such as Greengenes or SILVA to group the data based on taxonomic relationships. Two ecological indicators, α-diversity (e.g., the Shannon index) and β-diversity (e.g., the Bray-Curtis distance), can reveal changes in community organization. Complex statistical methods, such as LEfSe and associated network analysis, are used to identify species associated with disease status (Wang et al., 2023; Niu et al., 2021). PICRUSt and similar functional prediction tools infer potential metabolic processes from 16S rRNA data, thereby enhancing its utility by approximating metagenomic insights (Chen Z. et al., 2025; Savitri et al., 2025). Ultimately, integrating different types of omics data, especially metabolomics and transcriptomics, will facilitate a deeper understanding of the interaction between the host and gastrointestinal microorganisms. Collectively, these tools provide fundamental insights into bacterial colonization and survival in the gastric niche. When infected with *H. pylori*, the number of bacterial types will decrease. Specifically, spirochetes are enriched, whereas *Mycobacterium* and *Lactobacillus* are depleted (Wang and Li, 2021; Jiang et al., 2021). Different microbial characteristics have been found in gastric cancer. These characteristics are mainly composed of oral bacteria, such as *Neisseria* and *Prevotella*. These taxa may contribute to carcinogenesis by modulating immune responses and mucin production (Cervantes et al., 2021; Li et al., 2024). Medical interventions, such as gastrectomy and proton pump inhibitors, can significantly alter the body's microbial community. These changes are related to specific metabolic outcomes and healing pathways (Oosterlinck et al., 2023; Gutiérrez-Repiso et al., 2021).

In summary, 16S and metagenomic sequencing are two different methods that, when used together, provide a full picture of the organisms living in the stomach and how they function. Significant insights remain to be gained regarding how the microbiota influences gut health and disease through the continued refinement of analytical tools, including network analysis, functional prediction, and multi-omics integration. These advances will facilitate the development of microbiome-guided personalized therapies.

#### 2.3.2 Metabolomics approaches for characterizing the microbiota-metabolism axis

Metabolomics comprises a powerful set of analytical tools for the constituent molecules of the human body. Mass Spectrometry (MS) and Nuclear Magnetic Resonance (NMR) are the two main types. Ultra-sensitive MS, such as GC-/LC-MS, serves as a “precision detector” that detects metabolic signals, such as alterations in the saliva of pediatric patients with metabolic syndrome and certain chemicals in the blood of adults with *H. pylori* gastritis that underscoring the link between bacterial colonization, microbiota composition, and host metabolism. NMR, on the other hand, is the “steady scanner.” This technique facilitates highly accurate, non-invasive analyses that are particularly effective for elucidating tissue responses to pharmacological interventions (Sharma et al., 2025; He et al., 2024).

Integrating these metabolic “maps” with sequencing-based “species censuses” of the microbiome maximizes their utility. This integration has elucidated mechanisms, such as tryptophan metabolism, through which gut microorganisms modulate gastric inflammation and mucosal healing (Carneiro et al., 2022). It reveals how bacteria can synthesize short-chain fatty acids and other metabolites that modulate systemic lipid and energy metabolism remotely. The presence of *H. pylori* infection promotes hepatic lipid accumulation. Computational tools such as MetOrigin serve as a “GPS” to delineate the functional roles of chemicals and microorganisms by tracking their complex interactions with one another (Song et al., 2025; Fu et al., 2025).

Metabolomics deciphers the biochemical dialogue between the microbiota and the host. It facilitates the identification of pathogenic metabolites, therapeutic mechanisms, and ultimately, the function of the “microbiome-metabolite” axis.

#### 2.3.3 Future directions in multi-omics integration

Multi-omics analysis, which combines microbiome, metabolome, and transcriptome data, has enabled new approaches to fully understand how organisms respond to hypoxia, Microbiome investigations characterize structural alterations in microbial communities induced by hypoxia. At the molecular level, metabolomics clarifies these conclusions by showing that important microbial metabolites, such as short-chain fatty acids and bile acids, exhibit significant changes. In rat models of hypobaric hypoxia, similar metabolic changes are linked to ventricular hypertrophy, and treatments that target the microbiota only partially ameliorate these abnormalities. This underscores the critical role of microbial metabolites in the pathogenesis of disease. Transcriptomics provides mechanistic insights into this phenomenon by assessing how host genes respond to environmental changes. Studies have demonstrated that microbial colonization significantly influenced the transcriptional patterns in the hearts of mice exposed to intermittent hypoxia and hypercapnia. Combining multiple omics layers makes it easier to identify key microbial taxa, metabolites, and host genes that contribute to how organisms adapt to low oxygen levels. This offers a more comprehensive understanding than any single omics technique could in isolation. Finally, systems biology uses these data to construct interaction networks and computational models that explain how the “microbiome-metabolome-host” axis operates under hypoxic conditions. A multi-omics study, for instance, linked certain bacteria, such as *Bacteroides*, to alterations in the metabolism of short-chain fatty acids and bile acids, which helped explain a complete pathway leading to heart hypertrophy.

Through an integrated multi-omics analysis, systems biology enables a comprehensive understanding of the complex pathophysiology associated with hypoxia. In oncology, multi-omics integration has identified ACOT1 as a key molecular target through which piperine, a bioactive compound derived from long pepper, alters lipid breakdown in gastric cancer. This reveals a novel mechanism by which the microbiome may modulate tumor energy metabolism (Zhou et al., 2021). In summary, systems biology analyses greatly improve our understanding of both adaptive and pathogenic networks that work in hypoxic microenvironments. This provides a strong mechanistic basis for the development of targeted therapies, although the translation of these insights into clinical practice remains largely aspirational given the current state of evidence.

### 2.4 Future research directions and possible uses in the clinic

#### 2.4.1 Development of therapeutic strategies focused on the microbiota-metabolism axis

Modulating the interplay between microbiota and metabolism is a novel approach to treating gastric hypoxia. Hypoxia of the gastric mucosa promotes inflammation and tissue injury by disrupting the microbiota-metabolism equilibrium. Therapeutics targeting metabolic and microbial pathways can facilitate mucosal restoration. Flavonoids, such as those produced by lactic acid bacteria, can influence bacterial aggregation. This may promote the production of beneficial molecules, such as tryptophan and short-chain fatty acids. This prevents immune hyperactivation and strengthens the mucus barrier. Some therapeutic effects are related to improving metabolism and reducing inflammation (Singhal and Shah, 2020; Dong et al., 2025).

There is growing interest in modulators of specific metabolic pathways. Using amino acids to alter bile acid degradation (e.g., taurine-bound bile acids) and to produce polyamines may have a significant impact on the stomach microenvironment. Changes in bile acid levels activate FXR/TGR5 receptors, which control inflammation and energy balance. Tryptophan metabolites, such as indole compounds, function as essential signaling molecules that modulate immunological responses and maintain epithelial integrity through the aryl hydrocarbon receptor. Microbiome-targeted treatments that increase the production of beneficial tryptophan metabolites are new approaches to treat inflammatory and metabolic gastric illnesses (Singhal and Shah, 2020; Münzker et al., 2022).

Many studies have shown that metabolites are effective biomarkers, linking various bacteria, their metabolites, illness states, and medication outcomes. For example, changes in the ratio of *Klebsiella* to *Bacteroides* and amino acid catabolism in patients with gastric cancer are linked to the efficacy of neoadjuvant chemotherapy. This suggests that metabolomic analysis might facilitate the development of more tailored treatment plans in the future, pending prospective validation (Zhao et al., 2025). In the same manner, specific fatty acid profiles and changes in the variety of microbes in the feces of patients with *H. pylori* infections provide us with new tools to monitor the condition and assess how effectively treatments work.

In summary, several strategies have been proposed to address the hypoxic gastric environment, including probiotics, metabolic modulators, and plant-derived nanoparticles. These therapies aim to restore healthy microbial populations, regulate critical mucosal metabolic pathways, and convert metabolites into biomarkers that may eventually inform clinical decision-making, pending robust prospective validation. Future research should focus on understanding how microbes and metabolites interact under hypoxic conditions and on building the evidence base needed for the translation of these targeted strategies into clinical practice, acknowledging that most current findings remain preclinical or observational, with the goal of enhancing treatment outcomes for gastric diseases.

#### 2.4.2 Future directions toward individualized approaches and biomarker development

The metabolome and the microbiome can both elucidate how gastric pathologies evolve under hypoxic conditions. Specific microbial taxa undergo metabolic reprogramming and compositional shifts. These alterations influence disease phenotype and progression risk. In patients with gastric cancer, certain patterns of dysbiotic growth are associated with more aggressive tumors and worse prognosis. Associated factors include hypoxia, DNA damage, and immune suppression.

Hypoxia correlates with specific biochemical and microbiological markers that aid clinicians in assessing patient risk stratification and therapeutic response. Microbiome features should be incorporated into pharmacological models to predict drug efficacy and adverse effects. The results of this study enable the future development of biomarker-based tools that leverage both microbiome and metabolomic features. These tools may eventually support more targeted therapeutic approaches, although prospective validation is essential before clinical deployment, and could facilitate more accurate risk assessment.

Subtly yet significantly, hypoxia modulates the microbiota-metabolism axis in the stomach. Comprehensive understanding of this network necessitates the integration of data from multiple sources. Combining genomic, transcriptomic, metabolomic, and microbiome data provides a more comprehensive understanding of the interactions among the host, microbiota, and metabolites under hypoxic conditions. For example, combined transcriptional and metabolic analyses have identified important pathways such as the HIF-1α axis, glycolysis, lipid reprogramming, and microbiota-mediated immune regulation (Chen L. R. et al., 2025; Fu et al., 2025). In oncology, multi-omics further defines signaling axes, such as Ephrin-A3/EphA2 and YY1-CPT1C, that drive hypoxic metabolic plasticity and tumor progression, providing new targets for precise cancer therapy (Zhou et al., 2021).

Modulating the hypoxia-adrenergic axis with nanotechnology represents one avenue by which integrated omics data could be applied to drug development. Integrating multi-omics data enables patient stratification based on molecular profiles, enabling prediction of therapeutic response and more tailored drug selection—though these approaches remain conceptual without prospective interventional validation—such as modulating the microbiota, targeting specific metabolic pathways, and employing immunotherapy. Computational biology and systems medicine can translate complex omics data into algorithms that support clinical decision-making, representing a potential step toward dynamic, adaptive treatment regimens. By elucidating the mechanisms through which hypoxia drives gastric pathology, multi-omics integration technologies can guide the development of individually optimized therapeutics. However, this goal remains a long-term aspiration, as the evidence base is predominantly observational.

### 2.5 Limitations and challenges

The confounding effects of *H. pylori*, the influence of PPIs, sampling heterogeneity, and the difficulty in distinguishing between physiological and pathological hypoxia pose significant methodological challenges in studies of the gastric microbiome and hypoxia. These factors necessitate systematic consideration and control in future experimental designs.

First, the confounding effect of *H. pylori* is one of the most disruptive variables in gastric microbiome research. As a dominant colonizer of the stomach, *H. pylori* not only significantly reshapes the overall structure and relative abundance of the gastric microbiome but may also mask or amplify the true associations between other microbial communities and disease. In a large-scale case-control study involving 268 gastric cancer patients and 288 controls, Gunathilake et al. (2019) found that the relative abundance of *H. pylori* was significantly positively associated with gastric cancer risk (OR = 1.86, 95% CI: 1.17–2.97); however, this effect varied substantially before and after adjusting for *H. pylori* status in the regression model, suggesting that spurious associations may arise if this variable is not strictly controlled. Furthermore, *H. pylori* infection can induce local inflammatory responses in the gastric mucosa and alter gastric acid secretion, indirectly affecting the gastric microbiome and hypoxic microenvironment, thereby obscuring the true causal pathway. Therefore, in population-based studies, *H. pylori* infection status (determined by histology, rapid urease test, or breath test) should routinely be assessed and adjusted for in multivariate analyses, or *H. pylori*-positive and -negative subgroups should be analyzed separately during the study design phase to minimize confounding effects.

Second, the interference of proton pump inhibitors (PPIs) with the gastric microbiome cannot be overlooked. By strongly inhibiting gastric acid secretion, PPIs significantly raise intragastric pH, thereby promoting the colonization and proliferation of bacteria of oral and pharyngeal origin in the stomach, leading to significant changes in the α- and β-diversity of the gastric microbiome (Shi et al., 2019; Bruno et al., 2019). A study by Shi et al. (2019) demonstrated that in patients with gastroesophageal reflux disease (GERD) receiving PPI therapy, the relative abundance of oral-derived bacterial groups such as *Planococcaceae, Oxalobacteraceae*, and *Sphingomonadaceae* was significantly elevated in the gastric mucosal microbiota, while the abundance of the genus *Methylophilus* increased further in those with long-term PPI use. A systematic review by Fossmark and Olaisen (2024) further confirmed that PPI use leads to the abnormal proliferation of oral microbiota, represented by *Streptococcaceae, Veillonellaceae*, and *Haemophilus*, in the upper and lower gastrointestinal tracts. Bruno et al. (2019) also pointed out that PPIs induce dysbiosis of the gastrointestinal microbiota by creating a low-acid environment in the stomach, which may subsequently contribute to the pathogenesis of various gastrointestinal diseases. Therefore, in gastric microbiome studies, a history of PPI use must be included in the analysis as a key exclusion criterion or covariate; otherwise, the observed microbial differences may reflect drug effects rather than the characteristics of the disease itself.

Third, sampling heterogeneity is another technical challenge that cannot be overlooked in gastric microbiome research. Microbial communities in different anatomical regions of the stomach (such as the antrum, body, and cardia) exhibit significant spatial heterogeneity in composition and density. This heterogeneity is attributed to regional differences in gastric acid secretion, uneven *H. pylori* colonization, and variations in mucosal blood flow distribution. Additionally, the depth and size of biopsy samples, as well as the presence of mucus or blood, may influence sequencing results. Alwahaibi (2026) noted that morphological assessment is limited by inter-observer variability and sampling constraints, and that single-site biopsies are insufficient to fully capture the molecular heterogeneity and evolutionary dynamics of gastric microbiota. Therefore, it is recommended to adopt a multi-site, standardized biopsy protocol and collect at least two samples from the same anatomical site in the same patient to improve the reproducibility and representativeness of the results. Furthermore, factors such as pre-biopsy fasting duration, the volume of air insufflated during endoscopy, and the duration of the procedure must be strictly controlled to minimize procedural-induced technical bias.

Finally, the difficulty in distinguishing between physiological and pathological hypoxia is a core scientific issue that requires urgent resolution in studies of gastric mucosal hypoxia. Under normal physiological conditions, the gastric mucosa exhibits an oxygen gradient: the partial pressure of oxygen decreases from the mucosal surface to the deep glands. This physiological hypoxia is crucial for maintaining the gastric mucosal barrier function and stem cell homeostasis. However, under pathological conditions such as gastric cancer and chronic atrophic gastritis, rapid proliferation of tumor cells and abnormal angiogenesis lead to further deterioration of tissue oxygen supply, resulting in a pathological hypoxic microenvironment. Yashiro et al. (2026) compared the functional differences in gastric AGS cancer cells under cobalt chloride (CoCl_2_)-induced chemical hypoxia and 1% O_2_ true hypoxia conditions. They found that the regulatory patterns of the LPA receptor signaling pathway were markedly different under hypoxic conditions—the former upregulated LPAR1 and LPAR3 expression, while the latter downregulated these receptors, suggesting that chemically simulated hypoxia and true hypoxic environments differ fundamentally and cannot be simply equated. This finding underscores the necessity of carefully distinguishing whether the elevation of markers associated with gastric mucosal hypoxia stems from normal physiological oxygen regulation or tumor-specific pathological processes. Direct measurement of tissue oxygen partial pressure using oxygen probes, combined with multidimensional assessment of HIF-1α and its downstream target genes, and supplemented by rigorous histopathological controls, will help improve the accuracy of hypoxia classification.

In summary, the confounding effects of *H. pylori*, PPI use, sampling heterogeneity, and the difficulty in distinguishing between physiological and pathological hypoxia represent core challenges currently facing research on the gastric microbiome and hypoxia. Future studies should strictly control these variables in prospective cohort designs, adopt standardized, multicenter sampling protocols, and incorporate high-resolution technologies such as spatial transcriptomics and single-cell sequencing to more accurately elucidate the complex interactions between the gastric microbiome and the hypoxic microenvironment.

Fifth, the intrinsically low biomass of the gastric microbiome poses a pervasive but frequently underappreciated challenge. Gastric microbial loads are orders of magnitude lower than those of the colon, rendering sequencing datasets highly susceptible to contamination from reagents, endoscopes, and laboratory environments. Negative controls—including blank DNA extraction controls and no-template PCR controls—are inconsistently reported across studies, and when available, they frequently reveal contaminant sequences that overlap with taxa reported as genuine gastric residents. Computational decontamination tools such as Decontam, which leverage either the prevalence of taxa in negative controls or the correlation between sequence frequency and DNA concentration, should be routinely applied, and both raw and decontaminated datasets should be reported to allow independent verification. Furthermore, oral-derived taxa commonly detected in gastric samples may reflect true translocation from the oropharynx during swallowing, but could also represent contamination or transient passage; distinguishing these possibilities requires careful temporal sampling and source-tracking analyses.

Sixth, antibiotic exposure and dietary factors represent critical confounders that are rarely adequately controlled. Antibiotics—particularly the multi-drug regimens used for *H. pylori* eradication—produce prolonged and sometimes irreversible alterations in the gastric microbiome that may persist for months to years, yet many cross-sectional studies do not systematically record recent antibiotic use, risking the misattribution of antibiotic-induced dysbiosis to disease-related hypoxia. Similarly, dietary patterns profoundly influence the gastric microenvironment: high-salt diets promote *H. pylori* colonization and exacerbate mucosal inflammation, while polyphenol-rich diets modulate microbial composition and metabolite output. The near-absence of standardized dietary questionnaires and antibiotic exposure records in existing studies represents a significant gap. Prospective studies should, at minimum, collect detailed dietary histories and document antibiotic exposure within the preceding 6 months to permit meaningful stratification.

Seventh, technical variability in sequencing depth and analytical pipelines introduces substantial batch effects that compromise cross-study comparability. Shallow sequencing depths fail to detect low-abundance but functionally important taxa, while inconsistent sequencing depths across samples can generate spurious differences in alpha and beta diversity. Beyond depth, differences in DNA extraction kits, PCR primer selection, sequencing platforms (e.g., Illumina MiSeq vs. NovaSeq), and bioinformatic pipelines (e.g., QIIME2 vs. MOTHUR) collectively contribute to systematic batch effects that may outweigh biological signals. To address these issues, we recommend that studies report per-sample sequencing depth and adopt either rarefaction to a common depth or depth-corrected normalization methods. For cross-cohort meta-analyses, batch-correction tools such as ComBat or MMUPHin should be employed. Adoption of the consensus protocols proposed by the International Human Microbiome Standards consortium would substantially enhance reproducibility and facilitate meaningful integration of findings across independent studies.

## 3 Conclusion

This review systematically examines the hypoxia–microbiota–metabolism triad as an integrated pathogenic framework for gastric diseases, moving beyond the conventional pairwise paradigms that have dominated the literature. Rather than treating hypoxia, microbial dysbiosis, and metabolic reprogramming as independent variables, we argue that they constitute a self-reinforcing network in which each node amplifies the others: hypoxia remodels the microbiota, dysbiotic microbiota rewire metabolic output, and altered metabolites—succinate, lactate, depleted short-chain fatty acids, and dysregulated tryptophan catabolites—feed back to stabilize HIF signaling and perpetuate the hypoxic niche. This triadic model better accounts for the clinical observation that gastric pathology rarely resolves when only one axis is targeted, and it provides a mechanistic rationale for combination strategies that simultaneously modulate oxygen sensing, microbial composition, and metabolic pathways.

A critical insight emerging from this synthesis is the distinction between physiological and pathological hypoxia in the gastric mucosa. The stomach is constitutively hypoxic (mucosal pO_2_ ≈ 6 mmHg), and this physiological state is integral to barrier maintenance and stem cell homeostasis. Disease arises not from hypoxia *per se*, but from its pathological intensification—driven by *H. pylori* infection, inflammation, or neovascular dysfunction—which pushes the system past a tipping point where adaptive HIF signaling becomes maladaptive. Defining this threshold, and identifying the microbial and metabolic signatures that mark the transition, should be a priority for future research, as it would enable risk stratification before irreversible mucosal damage occurs.

Equally important, this review highlights the methodological challenges that currently limit the field. The confounding dominance of *H. pylori*, the pervasive effects of proton pump inhibitors, sampling heterogeneity across gastric anatomical sites, and the fundamental difficulty of distinguishing chemical from true hypoxia in experimental models collectively impede the disentanglement of causal relationships from epiphenomena. Addressing these challenges will require prospective cohort designs with standardized multi-site biopsy protocols, rigorous documentation of medication history, and the adoption of spatially resolved technologies—such as spatial transcriptomics and *in situ* metabolomics—that preserve tissue context rather than reducing it to homogenized averages.

The convergence of multi-omics integration with clinical data is beginning to translate this triadic framework into potential clinical applications that require independent prospective validation. Microbiome profiling combined with metabolomic signatures has already shown promise in predicting neoadjuvant chemotherapy response in gastric cancer and in stratifying patients by inflammatory risk. However, the current evidence base remains largely observational and cross-sectional. Longitudinal interventional studies—testing whether HIF inhibition, targeted microbiota restoration, or metabolic pathway modulation can interrupt the vicious cycle at specific nodes—are essential to validate the therapeutic implications of the triad model. Ultimately, we envision a future in which the hypoxia–microbiota–metabolism axis serves not merely as a descriptive framework, but as a basis for clinical decision-support, although this vision remains aspirational: guiding when to restore oxygen homeostasis, which microbial taxa to replenish, and which metabolic pathways to intervene upon—individually or in combination—for each patient's gastric disease trajectory.
